# Applying systematic review search methods to the grey literature: a review of education and training courses on breastfeeding support for health professionals

**DOI:** 10.1186/s13006-021-00373-5

**Published:** 2021-04-06

**Authors:** Ivette Navarro, Jose M. Soriano, Salomé Laredo

**Affiliations:** 1 Joint Research Unit on Endocrinology, Nutrition and Clinical Dietetics, University of Valencia-Health Research Institute La Fe, Valencia, Spain; 2 Food & Health Lab, Institute of Materials Science, University of Valencia, Paterna, Valencia, Spain; 3 Amamanta. Breastfeeding Support Group, Valencia, Spain; 4 Atomic Spectroscopy Section, Central Service for Experimental Research, University of Valencia, Burjassot, Spain

**Keywords:** Health professionals, Lactation courses, Programs, Review, Training

## Abstract

**Background:**

Currently, lactation training courses aimed at health professionals are important for informing and supporting mothers who are breastfeeding. In this review, we seek to analyze similarities and/or variations in course content, modes of delivery, costs, teaching style and learning strategies among courses. To our knowledge, a review of lactation training courses available worldwide is lacking. Thus, the aim of this review is to describe course models aimed at training health professionals in lactation support for mothers.

**Methods:**

Through searching grey literature, training courses were obtained from several directories, including the Alaska Breastfeeding Coalition, International Board of Lactation Consultant Examiners (IBLCE), International Lactation Consultant Association (ILCA), Lactation Education Accreditation Association and Approval Review Committee (LEAARC), World Alliance for Breastfeeding Action (WABA), World Health Organization (WHO), and United Nations Children’s Fund (UNICEF).

**Results:**

Descriptions of ten training programs were included in the final review. Our group found variations in costs, modes of delivery and duration among courses.

**Conclusions:**

Certified training for health professionals in lactation is a promising approach for increasing and protecting breastfeeding. Breastfeeding mothers might also benefit from specifically trained health professionals, yet, well-conducted research on such training courses is still required. The variability in the mode of teaching, tuition costs and course content in breastfeeding education programs around the globe must be kept in mind when considering providing training on the optimal competency for health professionals.

**Supplementary Information:**

The online version contains supplementary material available at 10.1186/s13006-021-00373-5.

## Background

This review will describe key components and possible variations among lactation training courses aimed at health professionals. Training health staff on lactation is of significant importance to help strengthen capacities and attitudes towards breastfeeding, increase mothers’ confidence in their ability to nourish through breastmilk and improve the health status of the most vulnerable population, infants [1]. The integration of lactation and breastfeeding promotion education into medical and other health professional curricula is a key strategy for achieving one of the goals of Healthy People 2020 [2]. Therefore, improving the knowledge, attitudes and skills of health professionals through lactation training courses can better assist women with breastfeeding [3].

The benefits of breastfeeding for both mothers and children makes breastfeeding an important strategy to protect public health [4]. For instance, breastfeeding can decrease the incidence of infectious diseases [5] and the risk of breast and ovarian cancers [6]. Extended breastfeeding influences epigenetic effects on infant metabolism [7]. The WHO and UNICEF [8] recommends exclusive breastfeeding during the first 6 months and ideally continuing breastfeeding along with complementary foods for at least 2 years. Annually, approximately 595,379 infant deaths are attributed to the cessation of breastfeeding. Optimal breastfeeding duration could also prevent 98,243 annual deaths by significantly reducing the risk of mothers developing certain cancers and type II diabetes [9].

The variety of internationally recognized in-person and online learning programs on lactation support can be a powerful tool to increase and improve breastfeeding rates [10]. Breastfeeding management demands implementing evidence-based learning at a local and internationally recognized level to improve lactation support skills when training health professionals and improving breastfeeding outcomes [11].

International lactation organizations have established a variety of courses ranging from classroom lectures to online courses to prepare students and health professionals to assist women with breastfeeding [12]. For instance, the Lactation Education Accreditation and Approval Review Committee (LEARRC) [13] is a leader in standards and guidance on lactation education and currently provides certified courses. LEAARC also works closely with the Commission on Accreditation of Allied Health Education Programs (CAAHEP) [14] to ensure the legitimate recognition of academic lactation programs comply with the International Board of Lactation Consultant Examiners (IBCLE) [15]. Three program pathways are available: i) for recognized health professionals e.g. midwife or breastfeeding support counsellors working at an approved organization e.g. ABA; ii) course needs for CAAHEP certification when students have concurrent or previous health science education. These students don’t need to be registered when undertaking the education (e.g. student midwives) due to that is part of their study curriculum and iii) approved IBCLC mentorship [16]. In fact, CAAHEP provides accreditation of lactation programs which must include both didactic and clinical requirements, while LEAARC evaluation recommends programs to be accredited and approve and recognized lactation courses and clinical internships [16]. Lactation Consultant professionals are prepared according to Standards (“the minimum requirements to which an accredited program is held accountable”) [17] and Guidelines (“descriptions, examples or recommendations that elaborate on the Standards”) in accrediting programs. The purpose of this review is to explore whether some course variations exist.

Forty hours is the minimum duration for a recognized BF education program according to the LEAARC website [16]. The 40 h does not include the IBLCE exam and certification which changed in 2017) and 45 h is mentioned on a training page providing information about LEAARC [18] In contrast, the IBLCE [15] focuses on an examination at the conclusion of 90 h of didactic education on lactation management. In addition, courses listed on the LEAARC [13] website and in the ILCA [19] directories also enable students in different countries and regions to find programs with 90 h of total training. A variety of programs also offer the opportunity to complete clinical experience [19]. Furthermore, ILCA is a member of the International Board-Certified Lactation Consultants (IBCLC) which also provides a list of programs available for students looking for a lactation management course in-person, through distance learning or a combination of both. According to the IBCLC [20] website, more than 60 coordinators can be found around the globe to assist in the certification process. IBLCE does not provide, accept or accredit lactation education for the certification examination and does not recommend or endorse any lactation education course [15].

The World Alliance for Breastfeeding Action [21] is another important resource that gives health professionals the opportunity to improve their knowledge and competency regarding breastfeeding guidance. Specifically, WABA organizes and promotes an annual workshop to prepares candidates for certification on breastfeeding but WABA does not provide a course on lactation training other than holding their annual workshop in Malaysia. The main purpose of the WABA workshop is to gather information from available recognized courses and to identify potential variations and/or similarities among these courses. Preparing health professionals through available online and in-person courses or a combination of both is very important. For instance, hospital training for health professionals is an effective strategy to improve knowledge, skills, and practices [3, 22].

In summary, the aim of this review was to identify training courses aimed at health professionals involved in lactation and breastfeeding promotion in order to identify the components of the course models offered worldwide.

## Methods

### Design

The grey literature search included different strategies such as the Google Advanced Search Engines and targeted websites and applied the Preferred Reporting Items for Systematic Reviews and Meta-Analysis (PRISMA) to ensure adequate reporting standards [23] (Table 1, see Additional file 1).

**Table 1:** Description of selected training courses

Title	Place	Duration	In-person or online learning	Course content	Tuition fee	References
Lactation Management Course	Alaska	90 h, 32 contact hours	Not included	Breastfeeding-update for health professionals. Breastfeeding: good for the baby. Better for women. Mother kangaroo: affection that saves lives. Parental care. Clinical course on breastfeeding management. Extended course of breastfeeding management	Not included	[24]
Lactation Course for Health Professionals	Australia	120 h	In-person and Online learning	Early days to 12 months, Anatomy & Physiology, Sociology, Psychology & Counselling, Endocrinology & Biochemistry, Pregnancy, Birth & Nutrition, Immunology & Pathology, Pharmacology & Toxicology, Prematurity / Technology, Public Health/ Ethical & Legal issues, Research.	$496 (750 AUD)	[25]
Clinical Lactation Management	Brazil	100 h	Online	Breastfeeding Benefits for the infant, woman and society, Real and alleged causes of early weaning, Psychophysiology and Lactation Techniques, Complementary food and return to work, Resolving the most common complications, Counseling - The Art of Listening.	$64 (R$281.6)	[26]
Lactation Consultant Training	Canada	108 h	In person	Anatomy and physiology of the infant and breast, Nutrition and Biochemistry, Immunology and infectious diseases, Pregnancy, Labor, Birth & 1st Week of Breastfeeding, Breastfeeding Techniques & Equipment, Not enough Milk & Premature Babies, Artificial Infant Milk & WHO Code, Counselling and Communication, Ethics and Breastfeeding, Pharmacology and Breastfeeding.	$1500 USD	[27]
Breastfeeding Consultant Training for Health Professionals	Denmark	90 h	In-person	3 modules – more information upon request	Not included	[28]
South Africa Certified Lactation Consultants (SACLC): Post- Graduate Lactation- specific Course	South Africa	100 h	In-person and online	Scientific aspects of Lactation, Nutrition, Immunology and Infectious diseases, pathological conditions that may affect breastfeeding conditions, environmental effects on breast milk, South African National Health Policy, HIV, WHO code, ESPGHAN and BFHI policies and implementation of it in South Africa, needs of a developing / developed country.	$760 (R11400)	[29]
WABA and the Infant Feeding Consortium	South East Asia- Malaysia	2 weeks	In-person	Components of Breastmilk, Anatomy, Positioning, Opportunities and Challenges, Management, Etiologies, Treatment Milk Supply, Pharmacology Milk Storage, Excessive Lactation, Weaning from Breast-feeding, Basic Concepts of Re-lactation, Induced Lactation, and HIV and breastfeeding.	$3500 USD	[21]
University Expert in Consulting and Advice on Breastfeeding	Spain	900 h	Online learning	Communication and Education in breastfeeding, Anatomy and Physiology, normal process of breastfeeding and feeding, Special situations and maternal difficulties, Special situations and maternal difficulties, Neonatal difficulties in breastfeeding, TFE, Sociology of breastfeeding, Legislation, research and health publish.	$976 (900 Euros)	[30]
Breastfeeding Specialist course	United Kingdom	120 h	In-person	Anatomy and physiology, milk composition, Position and attachment, Breast pathologies, Breast surgeries and management of undersupply, Counselling/communication skills and connection, Ankyloglossia/Tongue ties, Breastfeeding devices.	$1400 (£1080)	[31]
Lactation Counselor Training Course	United States	52 h	In-person	Breastfeeding management training, Practical skills, Time-saving hospital strategies, Strategies and tips for special circumstances, e.g. Down Syndrome, newborns affected by medications, and more, Communication skills to mothers in a way that encourages them to choose breastfeeding, Public Health/WIC strategies that work to increase breastfeeding success.	$685 USD	[32]

### Sample

**Fig. 1:**
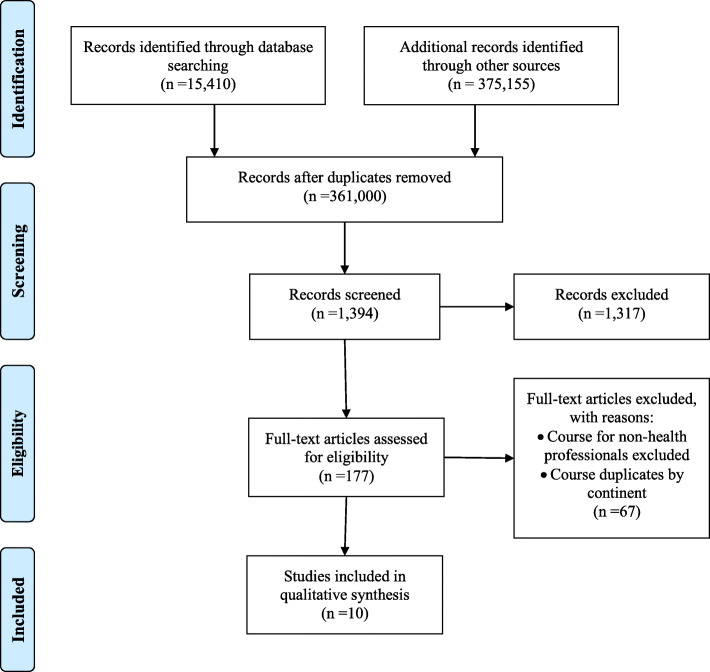
Flow chart applying the Preferred Reporting Items for Systematic Reviews and Meta-Analysis (PRISMA) [21]. This figure depicts the phases of screening and selection of courses and inclusion of the final synthesis

Figure 1 depicts the flow of screening and selecting the final courses for review. The following sites were searched as training providers: ILCA, IBLCE, LEAARC, WABA, Alaska Breastfeeding Coalition, Australasian Lactation, Aleitamento Brazil, Danish Committee for Health Education, Healthy Children Project Inc., Montreal Institute of Lactation Consultants (MILC) Inc., South Africa Certified Lactation, Spanish Foundation for Nursing Development, United Kingdom Breastfeeding Specialists, WHO and UNICEF. The search terms used were *lactation courses*, *breastfeeding*, *training*, *health professionals* and *programs*. Boolean operators were applied to assist in the search.

The inclusion criteria focused on lactation training courses for health professionals available from international and national web pages. Relevant peer-reviewed articles were also identified to select other pertinent information on the topic. Scientific articles were obtained from Cochrane Library, Medline, PubMed, and Google Scholar. The exclusion criteria involved articles that had no information about a course, or webpages without links to the training course. A total of 361,000 records were retrieved from the databases (Fig. 1), but only ten training courses were eligible for inclusion. The courses selected reported the formal aspects a review should contain such as, the critical reading of documents and programs, the stages of conducting a bibliographic review or the preparation of courses on lactation training for health professionals.

### Data collection

A systematic search of Internet sources was performed between December 2019 and February 2020 The search was not limited by the type of course or publication date. Furthermore, the importance of including any language was considered to evaluate courses worldwide. The three authors independently extracted the main information and data from each selected paper and course website. If a course or journal article contained a description of a training program, they were included for review. For example, courses were included if they focused on training certified health personnel responsible for guidance and education on lactation, and breastfeeding for woman after giving birth; such personnel included physicians, pediatricians, oncologists, nutritionists, midwives, and nurses among others [15]. From the grey literature systematic search an advanced Google search engine was implemented using previous search terms. A total of 361,000 related results for lactation training courses for health professionals was found. Godin et al. [33] criterion, which is based on several different search strategies such as the use of grey literature databases, customized Google search engines, targeted websites and consultation with contact experts were used to reduce bias. A total of 67 courses were extracted from internationally recognized programs with ten courses selected for review covering at least one course from each continent. Courses were excluded if they aimed at non-health professionals, or mother-to-mother support groups and trained volunteers. Other exclusions were applied to duplicate country-based programs. No restrictions were applied to language or program launch dates.

A form was prepared to extract data from the different training programs. The form helped identify who was being trained by which internationally recognized organizing body. The aim was to discover whether training took place in-person or via distance learning and to identify the content presented at each course. Our group attempted to obtain a description of the training including lectures, small group teaching, practical scenarios and use of other hands-on strategies. We also surveyed the length of each training program and attempted to identify the tuition cost of each course. The information collection identified the following descriptors: course title, country, duration, in-person or online learning, course content, tuition cost, sponsor program and website (Table 1).

### Data analysis

All information was independently analyzed by the three authors. The extracted information was divided among the three researchers who resolved discrepancies through discussion.

## Results

The list of webpages corresponding to ten lactation course programs is provided in Table 1. All tuition costs are in U.S. dollars. Foreign currencies were converted to U.S. dollars using the Google Finance Currency Converter online platform [34]. The final analysis included programs from Alaska [24], Australia [25], Brazil [26], Canada [27], Denmark [28], South Africa [29], South-East Asia Malaysia [21], Spain [30], the United Kingdom [31] and the United States of America [32].

Two programs were classed as distance or online learning, five involved in-person training, two offered a combination of both, and one did not specify the mode of learning delivery. Eight out of the ten course websites provided a list of topics and themes to be covered during the program; programs organized in Alaska and Denmark were excluded since not enough information was available for this review.

All program websites indicated a program’s duration which ranged from 52 h to 900 h. The tuition cost for eight programs was clearly indicated on the websites, while the two courses offered in Alaska and Denmark did not provide information on tuition costs. These costs ranged from as low as 64 U.S. dollars for the program in Brazil to 3500 U.S. dollars for the training workshop in Malaysia. Online training was cheaper than in-person learning. All ten programs indicated the course duration in either days or hours.

## Discussion

To our knowledge, this is the first systematic review of lactation training course content for health professionals. Our grey literature systematic search did not identify any articles that compared lactation training programs available to health professionals. The training courses included in our review were internationally recognized by organizing bodies such as LEAARC, IBLCE, ILCA and WABA. Most of these programs are organized and validated by regulatory bodies such as the CAAHEP.

Training programs can be organized in-person, online or through a combination of both, with potential variations within each learning platform. A potential variation of in-person training is the ability to interact with peers and the opportunity to use props and other teaching aids. Some studies have shown that e-learning can aid raising awareness among students [35]. Similarly, the mode of delivery varies. For example, online learning can provide the flexibility and convenience of completing the course at home. On the other hand, online learning may limit interaction between classmates and engagement in hands-on learning. Balogun et al. [36] demonstrated the efficiency of e-learning in improving awareness and positive attitudes toward breastfeeding among healthcare professionals. Consequently, variations among courses should be considered by health professional students.

A further important variation among the programs was training duration [37]. For example, the three-day Breastfeeding Consultant Training for health professionals may be more intensive than 12 h of lactation training over a 30-day period. It was also observed that the range of program’s duration varied from 52 h as the minimum for the U.S. Lactation Counselor Course, to 900 h at the university level in Spain. This observation shows that certain programs provide in-depth training over a longer duration and/or a more flexible time span to complete the course. Specific programs such as the ABC and the Danish Committee for Health Education program, did not identify tuition costs on their website and other web browsers. Tuition costs also varied widely among programs ranging from around 50 dollars to more than one thousand dollars. Furthermore, except for the Danish Committee for Health Education program which did not include a course description on its website, each program covered a range of topics and modules.

Training health professionals through various lactation programs has the potential to improve the management of breastfeeding and further improve breastfeeding outcomes [35]. Program variations potentially allow qualified health professionals to choose the program that best fits their needs. This flexibility is a key strategy for training health staff with breastfeeding guidance skills. Evidence has shown that an increase in the breastfeeding rates among women who received education and support from healthcare professionals can make even the slightest positive impact on maternal and infant health when compared to women who receive standard care [38]. Although courses for non-professional health workers were not the focus of this review, WHO [39] indicated the usefulness of trans-professional education where health professionals learn with, from and about non-professional health workers. Thus, training both professional and non-professional health workers might be of even greater importance for health-system performance [40].

### Limitations

As with any review, the search terms may have resulted in the omission of web pages. Furthermore, certain websites may have been missed because some countries may restrict access to their web pages.

## Conclusion

In summary, a further review of training courses and their various components is needed to identify and report variabilities and similarities among training programs currently available around the globe. The importance of training health care professionals on properly guiding and informing mothers on breastfeeding also remains a key component in promoting and protecting breastfeeding practices. Thus, future revision of these courses will further enable the proper implementation of courses that can prepare health professionals to aid lactating mothers.

## Supplementary Information

**Additional file 1: Table S1.** Details of the PRISMA checklist of the systematic review to the Grey Literature.

## Data Availability

Data sharing was not applicable to this article, as no datasets were generated or analyzed during the current study.

## Abbreviations

CAAHEP: Commission on Accreditation of Allied Health Education Programs
ILCA: International Lactation Consultant Association
IBLCE: International Board of Lactation Consultant Examiners
LEAARC: Lactation Education Accreditation Association and Approval Review Committee
UNICEF: United Nations Children’s Fund
WABA: World Alliance for Breastfeeding Action
WHO: World Health Organization

## References

[1] EU Project on Promotion of Breastfeeding in Europe (2004). Protection, promotion and support of breastfeeding in Europe: a blueprint for action.

[2] Centers for Disease Control and Prevention (2013). Strategies to prevent obesity and other chronic diseases: The CDC Guide to strategies to support breastfeeding mothers and babies.

[3] Zakarija-Grković I, Burmaz T (2010). Effectiveness of the UNICEF/WHO 20-hour course in improving health professionals’ knowledge, practices, and attitudes to breastfeeding: before/after study of 5 maternity facilities in Croatia. Croat Med J.

[4] Ma P, Brewer-Asling M, Magnus JH (2013). A case study on the economic impact of optimal breastfeeding. Matern Child Health J.

[5] Duijts L, Jaddoe VW, Hofman A, Moll HA (2010). Prolonged and exclusive breastfeeding reduces the risk of infectious diseases in infancy. Pediatrics..

[6] Chowdhury R, Sinha B, Sankar MJ, Taneja S, Bhandari N, Rollins N, Bahl R, Martines J (2015). Breastfeeding and maternal health outcomes: a systematic review and meta-analysis. Acta Paediatr.

[7] Pauwels S, Symons L, Vanautgaerden EL, Ghosh M, Duca RC, Bekaert B, Freson K, Huybrechts I, Langie SAS, Koppen G, Devlieger R, Godderis L (2019). The influence of the duration of breastfeeding on the infant’s metabolic epigenome. Nutrients..

[8] World Health Organization (WHO) & United Nations Children's Fund (UNICEF) (2009). Baby-friendly hospital initiative: Revised, updated, and expanded for integrated care.

[9] Walters DD, Phan LT, Mathisen R (2019). The cost of not breastfeeding: global results from a new tool. Health Policy Plan.

[10] Giglia R, Binns C (2014). The effectiveness of the internet in improving breastfeeding outcomes: a systematic review. J Hum Lact.

[11] Howett M, Lauwers J (2013). The standardization of lactation education to improve professionalism and patient care. J Hum Lact.

[12] Webber E, Watkins AL (2017). Evolution of a profession: the role of accreditation in lactation education. J Hum Lact.

[13] Lactation Education Accreditation and Approval Review Committee (LEAARC). Accreditation. https://www.leaarc.org/index.html.

[14] International Board-Certified Lactation Consultants (IBCLC). IBCLC Care Directory. http://www.ibclccare.org/directory.html.

[15] International Board of Lactation Consultant Examiners (IBLCE). Certification. http://iblce.org/certification.

[16] Commission on Accreditation of Allied Health Education Programs (CAAHEP). Questions About LEAARC Evaluation. https://www.leaarc.org/FAQ.html.

[17] Commission on Accreditation of Allied Health Education Programs (CAAHEP) & Lactation Education Accreditation and Approval Review Committee (LEAARC) Standards and Guidelines for the Accreditation of Lactation Consultant Education Programs. https://www.leaarc.org/docs/LactationConsultantStandars2018.pdf.

[18] Breastfeeding Outlook. https://breastfeedingoutlook.com/index.php?pageID=254.

[19] International Lactation Consultant Association (ILCA). Directory of lactation management courses. https://www.learning.ilca.org.

[20] International Board-Certified Lactation Consultants (IBCLC). IBCLC Care Directory. http://www.ibclccare.org/directory.html. Accessed 6 Jan 2021.

[21] World Alliance for Breastfeeding Action (WABA). Trainings & Conferences. https://www.waba.org.my/whatwedo/hcp/training.htm Accessed 6 Jan 2021.

[22] de Jesus PC, de Oliveira MIC, Fonseca SC (2016). Impact of health professional training in breastfeeding on their knowledge, skills, and hospital practices: a systematic review. J Pediatr.

[23] Moher D, Liberati A, Tetzlaff J, Altman DG, PRISMA Group (2009). Preferred reporting items for systematic reviews and meta-analyses: the PRISMA statement. Ann Intern Med.

[24] Alaska Breastfeeding Coalition (ABC). Lactation Management Course. https://alaskabreastfeeding.org/events/lactation-mangement. Accessed 6 Jan 20210.

[25] Australasian Lactation Courses. Lactation Courses for Health Professionals. http://www.breastfeedingspecialist.com/page5.htm. Accessed 6 Jan 2021.

[26] Aleitamento. Courses and seminars. Retrieved from Aleitamento. http://www.aleitamento.com/servicos. Accessed 6 Jan 2021.

[27] Montreal Institute of Lactation Consultants (MILC) Inc. Lactation Consultant Training. http://milc.ca/classes.html. Accessed 6 Jan 2021.

[28] Danish Committee for Health Education. Courses, education and conferences. http://sundhedsformidling.dk/kerneydelser/kurser-og-foredrag.aspx. Accessed 6 Jan 2021.

[29] South African Certified Lactation Consultants (SACLC) Course structure. http://www.salactationconsultants.co.za/About%20Us/aboutus.php. Accessed 6 Jan 2021.

[30] Spanish Foundation for Nursing Development (known as “Fundación para el Desarrollo de la Enfermería, FUDEN). Course: “University Expert in Consulting and Advice on Breastfeeding”. https://cursos.fuden.es/fuden/curso/394-experto-universitario-en-consultoria-y-asesoramiento-en-lactancia-materna. Accessed 6 Jan 2021.

[31] Breastfeeding Specialist. Info about the Course. http://www.breastfeedingspecialist.com/page5.htm. Accessed 6 Jan 2021.

[32] Healthy Children Project Lactation Counselor Training Course. https://centerforbreastfeeding.org/lactation-counselor-training-course/lactation-counselor-training-course. Accessed 6 Jan 2021.

[33] Godin K, Stapleton J, Kirkpatrick SI, Hanning RM, Leatherdale ST (2015). Applying systematic review search methods to the grey literature: a case study examining guidelines for school-based breakfast programs in Canada. Syst Rev.

[34] Morningstar for Currency and Coinbase for Cryptocurrency. Google Finance, https://www.google.com/intl/en/googlefinance/disclaimer. Accessed 6 Jan 2021.

[35] Hannula L, Kaunonen M, Tarkka MT (2008). A systematic review of professional support interventions for breastfeeding. J Clin Nurs.

[36] Colaceci S, Giusti A, Chapin EM, Bettinelli ME, De Angelis A, Zambri F (2017). E-learning to improve healthcare professionals’ attitudes and practices on breastfeeding. Breastfeed Med.

[37] Watkins AL, Dodgson JE, McClain DB (2017). Online lactation education for healthcare providers: a theoretical approach to understanding learning outcomes. J Hum Lact.

[38] Balogun OO, O'Sullivan EJ, McFadden A, Ota E, Gavine A, Garner CD (2006). Interventions for promoting the initiation of breastfeeding. Cochrane Database Syst Rev.

[39] World Health Organization (2013). Transforming and scaling up health professionals’ education and training: World Health Organization guidelines 2013.

[40] Frenk J, Chen L, Bhutta ZA, Cohen J, Crisp N, Evans T, Kistnasamy B (2010). Health professionals for a new century: transforming education to strengthen health systems in an interdependent world. Lancet.

